# Siglecs in Brain Function and Neurological Disorders

**DOI:** 10.3390/cells8101125

**Published:** 2019-09-22

**Authors:** Shoib Sarwar Siddiqui, Rachel Matar, Maxime Merheb, Rawad Hodeify, Cijo George Vazhappilly, John Marton, Syed Azharuddin Shamsuddin, Hussain Al Zouabi

**Affiliations:** Department of Biotechnology, American University of Ras Al Khaimah (AURAK), Ras Al Khaimah 10021, UAE; rachel.matar@aurak.ac.ae (R.M.); maxime.merheb@aurak.ac.ae (M.M.); rawad.hodeify@aurak.ac.ae (R.H.); cijo.vazhappilly@aurak.ac.ae (C.G.V.); jmarton@aurak.ac.ae (J.M.); azharuddin.syed@aurak.ac.ae (S.A.S.); hussain.zouabi@aurak.ac.ae (H.A.Z.)

**Keywords:** Siglecs, sialic acid, ganglioside, brain, neurological disorder, myelin, multiple sclerosis, Alzheimer’s disease, microglia, ITIM, ITAM

## Abstract

Siglecs (Sialic acid-binding immunoglobulin-type lectins) are a I-type lectin that typically binds sialic acid. Siglecs are predominantly expressed in immune cells and generate activating or inhibitory signals. They are also shown to be expressed on the surface of cells in the nervous system and have been shown to play central roles in neuroinflammation. There has been a plethora of reviews outlining the studies pertaining to Siglecs in immune cells. However, this review aims to compile the articles on the role of Siglecs in brain function and neurological disorders. In humans, the most abundant Siglecs are CD33 (Siglec-3), Siglec-4 (myelin-associated glycoprotein/MAG), and Siglec-11, Whereas in mice the most abundant are Siglec-1 (sialoadhesin), Siglec-2 (CD22), Siglec-E, Siglec-F, and Siglec-H. This review is divided into three parts. Firstly, we discuss the general biological aspects of Siglecs that are expressed in nervous tissue. Secondly, we discuss about the role of Siglecs in brain function and molecular mechanism for their function. Finally, we collate the available information on Siglecs and neurological disorders. It is intriguing to study this family of proteins in neurological disorders because they carry immunoinhibitory and immunoactivating motifs that can be vital in neuroinflammation.

## 1. Introduction

Glycosylation is vital for normal brain function and an alteration in this process may lead to nervous disorders and death [1,2]. Glycosylated proteins, carbohydrate moieties, and lectins have been studied in brain function and neurological disorders [1,2]. One of the types of glycosylation that is abundantly found in the brain is sialylation. Sialic acid is a nine-carbon monosaccharide that is present at the terminal end of glycoproteins and glycolipids in higher invertebrates and all vertebrates [3]. Sialic acids (Sia) are also called “neuraminic acids” and this nomenclature is based on their discovery. In 1936, Gunnar Blix isolated them from saliva and named them Sialic acids (the Greek word for Saliva). In 1941 Ernst Klenk isolated them from brain glycolipids and named them neuraminic acid for the neurons in brain [3]. The role of sialic acid in brain development and function is compiled in an excellent review by Schnaar et al. [4]. Siglecs, which are cell surface receptors that bind sialic acids, are abundantly found in the immune cells [5].

### The Siglec Family

The discovery of Siglecs dates back to 1990 when CD22 (Siglec-2), B cell specific protein’s cDNA was cloned, and it was observed that this protein carries many immunoglobulin-like domains [6]. One year later, it was found that CD22 binds with sialylated glycoproteins thus revealing its role as sialic acid binding lectin [7]. Another Siglec, mouse macrophage specific receptor/sheep erythrocyte receptor (original name- sialoadhesin/Siglec-1) was purified and characterized from mouse spleen. It was found that it can agglutinate sheep and human erythrocytes in sialic acid-dependent manner [8]. Subsequent studies were carried out that identified and characterized Sialoadhesin, CD22, and myelin-associated glycoprotein (MAG/Siglec-4) as immunoglobulin-like domains containing the sialoadhesin family [9,10]. Finally in 1998, Paul Crocker and Ajit Varki with others, named this family of proteins “Siglecs” which stands for sialic acid binding immunoglobulin-type lectins [11].

**Figure 1:**
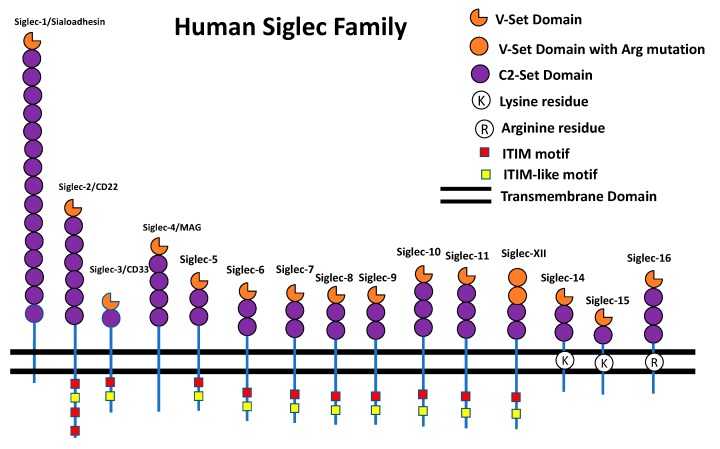
The human Siglec family receptors. The human Siglec receptors are expressed on the immune cells and have Ig-like extracellular domain. They carry one extracellular V-set domain that binds with sialic acid ligands. Beneath V-set domain these receptors carry different numbers of C2-set domains. Siglec-3 and Siglec-15 have only one C2-set domain while Siglec-1 has 16 C2-set domains. As an exception, Siglec-XII has two V-set domains but both of them do not have critical arginine and does not bind with sialic acid ligand. Most of the Siglecs have ITIM and ITIM-like domain that facilitates inhibitory signal to the cells. Siglec-14, -15, and -16 have positively charged residue in the transmembrane domain that recruit Dap12 and facilitate activating signal to the cells.

**Figure 2:**
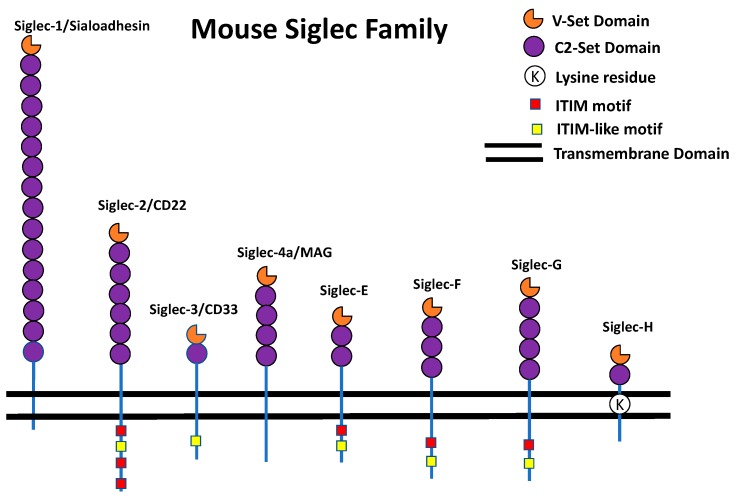
The mouse Siglec family receptors. In mouse and human, there are some Siglec receptors that are conserved such as Siglec-1, Siglec-2, and MAG while others are rapidly evolving and do not have true ortholog between human and mouse. These rapidly evolving Siglecs in mouse are CD33, Siglec-E, -F, -G, and -H. The outermost N terminal domain has V-set domain and below the V-set domain has C2-set domains similar to human Siglecs. In the intracellular domain there is ITIM and ITIM-like motifs.

Siglecs are type I transmembrane receptors that bind sialic acids. Siglecs are predominantly expressed on the surface of immune cells [12]. The human and mouse Siglecs are shown in Figure 1 and Figure 2. The human Siglecs family contains 15 members (Figure 1) that are basically classified into two groups: Evolutionary conserved Siglecs and rapidly evolving Siglecs. As the name suggests, the evolutionary conserved ones have the true orthologues present in mammalian species. The members of conserved Siglecs are Siglec-1, CD22, MAG, and Siglec-15 (Figure 1). The rapidly-evolving ones do not exist as true orthologues in the mammalian species and more commonly called CD33-related Siglecs. In primates, the members of the CD33-related Siglecs are CD33 (Siglec-3), Siglec-5-14, Siglec-16, and Siglec-17 (Figure 1) [5,12,13]. In mice, the Siglecs family contains 8 members (Figure 2). Siglec-1, Siglec-2, and MAG have true homologs in humans and mice. Siglecs are a rapidly evolving gene family. Due to this reason, many Siglecs do not have true homologs present between humans and mice. Based on the expression pattern and function there is annotation of some mouse and human Siglecs as functionally equivalent paralogs. Siglec-9 is functionally equivalent to Siglec-E whereas Siglec-8 is functionally equivalent to Siglec-F [5,12,13]. The mouse and human CD33 are completely different. They have different intracellular domains, different expression patterns, and bind to different ligands. Therefore, the studies that are carried out on mouse CD33 can usually not be extrapolated to human CD33 and vice versa [14].

Siglecs carry immunoglobulin like extracellular domains, a single pass transmembrane domain and an intracellular domain (as shown in Figure 1 and Figure 2). The N-terminus outermost immunoglobulin domain is called a V-set domain while subsequent immunoglobulin domains are called a C2-set domain. The V-set domains bind sialic acid while the C2-set domain does not have binding sites for sialic acid [5,12,13]. The extracellular V-set domain carries a critical arginine residue for interaction with sialic acid [5,12,13]. The intracellular domain has immunoreceptor tyrosine-based inhibitory motifs (ITIM) and ITIM-like signaling motifs. Upon ligand binding, these motifs get phosphorylated by Src family kinases and lead to intracellular signaling involving Src homology region 2 domain-containing phosphatase-1 (SHP-1) and Src homology region 2 domain-containing phosphatase-2 (SHP-2) recruitment [5,12,13]. These inhibitory signaling events may reduce the inflammatory response by blocking the MAP kinase (MAPK) signaling cascade. Thus, it acts like a brake for the inflammatory signaling pathways [5,15]. Contrary to this, there are some Siglecs that carry a positively charged residue in the transmembrane domain that recruit DNAX-activation protein of 12 kDa (DAP12) [16,17,18,19,20]. The DAP12 protein carries immunoreceptor tyrosine-based activating motifs (ITAM) that enhance the MAP kinase signaling cascade and lead to activation of the immune cells. Thus, inhibitory and activating Siglecs signal through opposing motifs and lead to opposite effects on the activation of immune cells [16,17,18,19,20]. Some of the Siglecs exist as paired receptors as they carry the same extracellular domain, but their transmembrane domain or intracellular domain generate opposite functions. Thus, they bind with the same ligand but one of them generates inhibitory signals while other generate activating signals to the cells. This is very important for the fine tuning of the immune response [21,22]. The role of paired receptors in the mouse models of cancer, inflammation, and bacterial infection have been studied recently [22,23]. There are also recent reports on the expression of Siglecs in non-immune cells. Siglec-6 was reported to be expressed on human placental trophoblast [24], Siglec-XII on epithelial cells [25], Siglec-5/14 on amniotic epithelium [21], Siglec-7 on pancreatic islets [26]. Siglec-3/Siglec-11/MAG in the brain tissue [27,28,29,30], and Siglec-9, -10, -11, and -16 are present in female reproductive tract [31,32]. While there are a plethora of literature and reviews available on Siglecs and immune cells, reviews compiling non-immune function of Siglecs are scarce. This review is focused on the role of Siglecs in brain function and neuronal disorders.

## 2. Siglecs and Their Role in Neurological Disorders

### 2.1. General Biology of Siglecs That are Expressed in the Brain

Sialoadhesin (Siglec-1, CD169) belongs to the category of conserved Siglecs, which has true orthologs present among mammalian species. It is basically a macrophage restricted glycoprotein which has an apparent molecular weight of 200 kDa. Sialoadhesin is the largest member of the Siglec family. It has 16 C2-set domains, one V-set domain, a transmembrane domain and cytosolic tail (Figure 1) [33]. Interestingly, the cytosolic tail of Sialoadhesin does not have any signaling motif. The sequence homology between mouse and human Sialoadhesin is 72% and it has a binding preference for α-2-3 linked sialic acid over α-2-6 linked sialic acid. The binding and expression pattern of mouse and human sialoadhesin are very similar. They are found on tissue macrophages and mostly absent from the surface of monocytes and other peripheral blood leukocytes [33]. The absence of ITIM motifs and preference of α-2-3-sialic acid binding led to the hypothesis that Sialoadhesin’s function is clearance of sialylated pathogens [34].

CD22 (Siglec-2) is usually expressed on B cells. The apparent molecular weight of CD22 is around 140-kDa and it has 6 C2-set domains and one V-set domain. In the intracellular domain it has 3 ITIM and one ITIM-like domain (Figure 1). CD22 functions as a co-receptor of B-cell receptor (BCR) and it regulates the B-cell response upon inflammation [35]. A cross-linking of CD22 and BCR upon antigen binding, stimulates the tyrosine phosphorylation of CD22 and further downstream signaling is mediated by associated phosphatases. A loss of CD22 leads to hyperactivation of the B cells and may lead to autoimmune disorders [35].

CD33 (Siglec-3) was discovered in 1983 when a set of monoclonal antibodies detected a 67 kDa glycoprotein on the surface of myelomonocytic cells in the hematopoietic system [36]. Later, CD33 was characterized as a member of sialic acid binding adhesin molecules and the recombinant soluble form of CD33 (CD33-Fc) was shown to preferentially bind Neu5Acα2-3Gal in N- and O-glycans [37]. Expression analysis found CD33 to be expressed in monocytes, microglia, a subset of B cells and activated T and NK cells [38,39]. CD33 is a canonical member of the CD33-related Siglec family and the smallest member of the family. It has one V-set, one C2-set domain, a single pass transmembrane domain and an intracellular domain (Figure 1) [36,37]. There are two splice variants of CD33: the short form of CD33 known as CD33m (D2-CD33) or the long form of CD33 known as CD33M [38].

MAG/Siglec-4 was discovered as a central nervous system (CNS) myelin glycoprotein. It was proposed that this protein is involved in mediating the interaction between glial and neuronal cells and is important for myelin sheath formation [40,41]. The apparent molecular weight of MAG is 100 kDa and 30% of its weight is constituted by glycosylation. The MAG cDNA was first cloned in 1987 [42]. Surprisingly, it was found to perfectly match another cloned cDNA p1B236 [43]. It was concluded that MAG and ps1B236 are the same. MAG binds with α-2-3-linked sialic acid, which is a part of many neuronal gangliosides [44]. The critical arginine in the V-set domain of MAG that interacts with sialic acid is present at 118th position in the N-terminal domain [44,45]. MAG is present in both CNS and peripheral nervous system (PNS) but the actual quantity of MAG with respect to total protein content is only 1% in CNS and 0.1% in PNS [46]. There are two splice forms of MAG, large (p72) and small (p67), which differ in their cytoplasmic domain. p67 carries 10 different amino acids and lack 54 amino acids that are present in p72 [46].

Siglec-11 was discovered and characterized in 2002 by the Varki group [47]. It specifically binds to α-2-8-linked sialic acid and is mainly expressed in peripheral blood leukocytes, macrophages and microglia [47]. It is an inhibitory Siglec that interacts with SHP-1 and SHP-2 upon ligand binding [47]. Siglec-11 is expressed in microglia of the human brain and a soluble recombinant form of Siglec-11 has shown to bind ligands in brain. Interestingly, Siglec-11 is not expressed in the brains of great apes [48,49].

Siglec-E was discovered in 2001 in a yeast two-hybrid screen using tyrosine phosphatase SHP-1 as bait [50]. It is the most abundant Siglec expressed on the surface of innate immune cells in mice [51]. Expression analysis showed that Siglec-E is expressed on the cell surface of neutrophils, monocytes, macrophages, dendritic cells and a subset of natural killer cells [51,52]. It is a disulfide-linked dimer on the cell surface, which has 2 C2-set domains and one V-Set domain (Figure 2) [51]. Siglec-E^−/−^ and transgenic chimeric Siglec-E16 (mouse with extracellular domain of Siglec-E and the transmembrane and intracellular domain of Siglec-16) mice have been used to decipher the role in bacterial infection, inflammation and cancer [22,23,53,54,55]. The functionally equivalent paralog of Siglec-E in humans is Siglec-9. Siglec-E preferentially binds to α-2-8-linked sialic acids which are highly abundant in brain on both NCAM and as shorter chains on gangliosides [52]. Interestingly, upon Lipopolysaccrides (LPS) stimulation, Siglec-E alleviates Toll-like receptor (TLR) signaling and helps resolve inflammation. Along with other Siglecs, Siglec-E can bind to the sialic acids of TLR and a sialidase treatment can disrupt this interaction, thus facilitating inflammation [56,57,58,59]. Siglec-E is found to be expressed on cultured mouse microglia [60].

Siglec-F is a murine Siglec which is found in high abundance on eosinophils. Earlier studies predicted that Siglec-5 is probably the true ortholog of Siglec-F, but there is only homology with the extracellular domains of Siglec-F [61]. There is no true ortholog of Siglec-F in humans, but the functionally equivalent paralog of Siglec-F is Siglec-8. Siglec-F preferentially binds to α-2-3-linked sialic acids and also recognizes 6′-sulfo-sLe^x^ [62]. It carries three C2-set domains, a V-set domain and an intracellular ITIM and ITIM-like motif (Figure 2) [62]. Siglec-F is expressed by microglial cells in the mouse brain [63].

Siglec-H is a member of the mouse specific CD33-related Siglec family, whose expression was found in plasmacytoid dendritic cells and it signals via DAP12. Therefore, it generates an activating signal to the cells [64]. Siglec-H is found to be expressed in microglia when activated with interferon-γ or polarized in M1 subtype. A comprehensive study was carried out to find the protein expression of Siglec-H in microglia. It was demonstrated that Siglec-H is expressed in microglia at all stages of embryonic development and adulthood. The expression of Siglec-H in microglia was not changed with inflammation or injury [65].

### 2.2. Role of Siglecs in Brain Functions

A study published in the early 1990s showed that a factor in serum or plasma regulates the expression of Siglec-1 (Sialoadhesin/CD169) in microglia. Microglia populations that reside behind the blood–brain barrier do not express sialoadhesin, whereas the ones that are close to the blood–brain barrier do express sialoadhesin. This observation pinpoints the relevance of a factor from plasma protein that induces the expression of Siglec-1 in microglia. Additionally, injuries to the CNS that expose these microglia to plasma protein further enhance the expression of Siglec-1 in microglia. This in vivo observation was the first study where Siglec-1 expression in microglia was observed and the mechanism was deciphered [66].

In a study by Mott et al., it was shown using a co-culture system with neurons and microglia that CD22 (Siglec-2) causes a reduction in the inflammatory effects of microglia [67]. The neurons were shown to express CD22 at the mRNA and protein level and a soluble form was released from cells which was responsible for decreasing the inflammatory effects of microglia [67]. In a recent study, the authors identified CD22 as a modifier of microglial phagocytosis in aging brain [68]. Microglia in the brain are usually involved in the clearance of aggregated proteins and cellular debris. However, in the aging brain, this function of microglia is hampered. In a CRISPR-Cas9 screen the authors identified CD22 as a master regulator of this function. A long-term delivery of blocking antibody against CD22 led to reversal of this phenotype in the aged brain of mice [68].

The critical function of MAG (Siglec-4) in the brain was highlighted by a study where this Siglec was found to inhibit neurite outgrowth and lead to axon growth collapse. MAG functions as a transmembrane protein and also as a soluble protein known as dMAG [44,46]. In the early studies, MAG was identified as promoter of neurite growth in dorsal root ganglion (DRG) or spinal cord cultures. Treatment with anti-MAG antibody led to rescue of this phenotype [69]. Additionally, under embryonic conditions MAG enhances the neurite outgrowth [70]. However, contrary to two simultaneously published pioneering studies in 1994 [71], it was established that MAG inhibits the neurite outgrowth [72]. The conflicting results of MAG on neurite outgrowth is mainly due to the difference in the developmental stages of the neurons on which the experiments were performed. The inhibitory role of MAG on neurite outgrowth is extensively studied because of its potential to regenerate neurons upon injury [72]. This phenotype was rescued by a treatment with anti-MAG antibody and recombinant MAG was a potent inhibitor of axon regeneration. Although it was shown in vitro in cerebellar and DRG neuron, it was proposed that the same mechanism might be happening in vivo as well [46,71]. With the previous findings it was clear that mammalian adult axons have little or no ability to regenerate after an injury, but the neonatal axons do regenerate well. One of the factors responsible for this is thought to be MAG present in myelin of CNS and PNS [46]. Although these findings were informative, an endogenous factor that is involved in this phenotype had not been found. Therefore, another study identified cyclic adenosine monophosphate (cAMP) as an endogenous factor that dictates the axonal regeneration [73]. It was observed that the cAMP levels were higher in young neurons as compared to older neurons. Moreover, an inhibition of cAMP downstream effector protein PKA (protein kinase A) inhibits the neuronal regeneration. Conversely, an elevation of cAMP in older neurons leads to blockage of inhibition of neurite outgrowth [73]. Thus, the neurite outgrowth inhibition by MAG involved downstream partners in the form of cAMP and PKA [74]. These findings were highly relevant in terms of their identification of factors responsible for inhibition of neurite outgrowth, but they also sparked a lot of controversies. The main controversy came from the finding that MAG^−/−^ mice have normal neurite outgrowth [75]. This actually does not negate the previous findings about MAG, but it certainly points out towards other factors present in myelin that are involved in the phenotype.

To further reinforce the idea that MAG is inhibitory in axonal regeneration, the soluble dMAG and chimeric MAG-Fc were used in vivo. As expected, dMAG and MAG-Fc were found to be potent inhibitors of axonal regeneration. Moreover, the regeneration was completely neutralized upon immunodepletion of dMAG or when a MAG antibody was used [76]. Further work has also supported the idea that dMAG diffuses through the CNS and affects the neurite outgrowth even when there is no physical contact between the neuron and myelin [74,76,77].

The most important microglial inhibitory motif carrying Siglec is Siglec-11 that interacts with the neuronal glycocalyx and alleviates microglia neurotoxicity [78]. The ligand for Siglec-11, PSA-NCAM (Polysialylated neuronal cell adhesion molecule) is expressed on both microglia and neurons but only neuronal expression of ligand (PSA-NCAM) is important for suppression of immune response by microglia. An ectopic expression of Siglec-11 in murine microglia cell line led to reduction of LPS-induced transcription of pro-inflammatory genes, marked decrease in phagocytosis and microglial cytotoxicity [79]. An activation of ITAM motif carrying receptors leads to activation of the cells and neuroinflammation [78]. Therefore, counteracting inhibitory ITIM motif carrying receptors are vital for maintaining homeostasis.

The expression of Siglec-E in cultured microglia leads to inhibition of phagocytosis of neural debris and alleviates the production of superoxide radicals upon stimulus. Furthermore, in co-culture conditions of microglia and neuron, Siglec-E was found to be neuroprotective and this phenotype was dependent on sialic acid [60]. Siglec-F is expressed by microglial cells and it interacts with neuronal sialic acid and is believed to affect neuronal integrity and morphology of microglia [63]. Using a co-culture system of microglia and neuronal cells, the treatment with endoneuraminidase and α-neuraminidase led to reduction of neurite integrity and number of neuronal cell bodies. Moreover, the Siglec-F expression on the microglia cell was also reduced with this treatment of neuraminidases. Overall, this study points toward the role of sialic acid and Siglec-F receptor in protection of neuronal integrity in case of neurogenerative diseases [63]. Neuraminidases are the enzymes (also called as sialidase) that cleave the sialic acid and sialic acid is ligand for Siglecs. We also want to point out that neuraminidases are also highly abundant in brain where they regulate the sialic acid expression. The function of neuraminidases in CNS function is discussed in a recent review by Pshezhetsky and Ashmarina [80]. Siglec-H expression in microglia facilitates the phagocytosis of mouse glioma cells in culture. This phagocytosis of glioma cells by microglia is dependent on Siglec-H and Dap12 [81]. The role of Siglecs in brain functions and disorders is summarized in Table 1.

### 2.3. Role of Siglecs in Neurological Disorders

In the neurodegenerative disease ceroid lipofuscinosis (CLN), the role of Siglec-1 (Sialoadhesin/CD169) has recently been deciphered. In two mouse models of CLN3, Ppt1^−/−^ and cln^−/−^ the expression of Siglec-1 was upregulated in microglia. To understand the role of Siglec-1 in CLN3 pathology, the two knockout mouse models were cross-bred with Siglec-1^−/−^ mice to generate Ppt1(^−/−^)Sn(^−/−^) and cln (^−/−^) Sn(^−/−^). In both these double knockouts, the neurodegeneration of the CNS was reduced, lifespan of mice was extended and the number of M1 polarized microglia were also reduced [82]. Additionally, Siglec-1 interacts with sialic acids that are heavily expressed in prions and thus may also play a role in prion diseases A prion protein can trigger the abnormal folding of the normal brain proteins and their pathology might involve spread from the secondary lymphoid organ to brain [83]. The spread of prion misfolding and aggregation to the brain can cause neurodegeneration and ultimately death. A hypothesis pertaining to this depicts Siglec-1 expressed by macrophages of lymph node interacting with sialic acids expressed in prions. However, in a Siglec-1 knockout mouse the hypothesis was rejected because prion pathogenesis was not different between the knockout and WT mice [84]. Another study showed that in the wild type mouse, Siglec-1 is rarely detected in CD11b+ cells, however the proteolipid protein (PLP) overexpressing mice that are demyelinated, almost all CD11b+ cells show Siglec-1 expression. PLP overexpressing mice also displayed elevation of CD8+ T cells and CD11b+ macrophages in the CNS. Interestingly, a double mutant where PLP is overexpressed and Siglec-1 is knocked out, the elevation of CD8+ cells and CD11b+ was restored [85].

Many Genome Wide Association Studies (GWAS) pointed out CD33 (Siglec-3) locus as a risk factor for late onset Alzheimer’s disease (LOAD) [86,87,88,89]. The molecular mechanism for the role of CD33 is not fully understood. It has been proposed that CD33 (an inhibitory Siglec) is expressed on microglia and interacts with sialylated amyloid plaques, which reduces the ability of microglia to phagocytose the amyloid plaques [27,90,91]. Thus, the overall clearance of amyloid plaque is reduced, and Alzheimer’s disease progresses more. It has also been proposed that the long form of CD33 is pathogenic whereas the short form is protective [90,91,92,93]. While deciphering the sub-cellular localization of both splice variants it was found that the long form stays at the cell surface while the short form goes to the peroxisome. The relevance of this observation was explained by the evolutionary theory of “Less is More” [94]. According to this theory, the short form that goes to the peroxisome reduces the overall burden of CD33M on the cell surface and thus alleviates the pathogenesis of Alzheimer’s disease [94]. Although there are other studies which show that CD33m (short form of CD33) is also present on the cell surface but the reason for this difference in observation could be the cell type that was used for the study [38,90]. In CD33m transfected cells, the protein appears to be present on the cell surface but the endogenously expressing CD33m cultured cell lines/primary cells show the expression in peroxisomes [38,90,94]. The role of CD33 in microglia and Alzheimer’s disease is extensively discussed in a comprehensive recent review [27].

In aged MAG^−/−^ (Siglec-4a^−/−^) mice, it has been observed that both myelin and axons are degenerated. Thus, this experiment in 8 months old mice refuted the notion that MAG is not involved in myelin sheath development in the PNS [95]. MAG and dMAG are highly relevant in cases where myelin sheath is disrupted such as multiple sclerosis. In multiple sclerosis, the neuronal degeneration is observed first, before the obvious myelin disruption and this could be due to the fact that dMAG might be acting before the collapse of myelin [74,96]. Due to obvious role of MAG in neuronal regeneration and multiple sclerosis there is a thrust to use it as a therapeutic target and GlaxoSmithKline has shown special interest in this molecule. The studies on the regeneration of neurons has been very fruitful in diseases such as stroke. A humanized monoclonal antibody (GSK24932) against MAG has been used in squirrel monkeys and clinical trials in treating strokes [97].

In 75% of benign monoclonal gammopathy patients, IgM antibodies react with MAG, sulfoglucuronyl glycosphingolipid (SGPG) or other glycoproteins of peripheral nerve [98]. Benign monoclonal gammopathies occur in around 1% of normal population of over 50 years of age and it is 10 times more frequent in patients with polyneuropathy [98]. IgM monoclonal antibody mediated gammopathy is the most abundant paraproteinemic neuropathy. The reactivity of IgM that leads to this neuropathy is dependent on the carbohydrate moieties of MAG [99]. The IgM antibody binding to MAG has also shown specificity to monosialoganglioside (GM1), the ganglioside GD1a and has been identified as SGPG. As discussed before, MAG is expressed in both CNS and PNS, but SGPG is expressed only in the PNS [98]. Therapeutic intervention with anti-MAG neuropathy is focused on depleting the monoclonal B cell population and reducing the concentration of antibody [98]. Rituximab, a monoclonal antibody against CD20 has shown promising results against this neuropathy [100]. The function of rituximab is through the suppression of IgM antibodies as well as anti-MAG antibodies and induction of regulatory T cells [100]. The role of Siglec-11 in neurological disorder is not well established. However, in the brain it interacts with polysialic acid (PSA), which is implicated in neurological disorders such as bipolar disorder, schizophrenia, and autism [4,101,102]. We have summarized the role of Siglecs in brain functions and disorders in Table 1. 

## 3. Conclusions

This review compiles the studies pertaining to brain functions and disorders which includes published literature related to microglia, demyelination and neuroinflammation. The role of few of the Siglecs has been extensively studied in immunity but overall the Siglec family has been less explored in neuroinflammation. The reasons for this could be lack of tools available to study them, non-expression of these Siglecs in brain, species-specific expression only in humans, or difficulty in the study of these Siglecs. One of the common problems in studying Siglec is the lack of monoclonal antibodies. However, recent efforts have been made to generate and characterize monoclonal antibodies against Siglecs [51]. More efforts have to be made to develop new blocking, non-blocking Siglec antibodies, and new mouse models to study the pathogenesis of brain disorders and Siglecs. An easy approach that has been widely used and should be used in future is the isolation of specific cell types from human brain samples or mouse samples and whole transcriptome analysis to pinpoint the Siglecs that are expressed in different cell types of CNS and PNS. One of the promising aspects of Siglec studies is the use of brain organoid cultures [103]. This will give impetus to studies pertaining to Siglec research and may pave the way for therapeutic intervention. With the development of technology and biological research we will be able to decipher the new role of Siglecs further in neuroinflammation, blood–brain barrier, and nervous disorders.

**Table 1:** Role of Siglecs in brain dysfunction.

Siglec Name	Alternate Name	Major Role	Major Role in Brain Disease/Disorder	Animal Model Exist	References
Siglec-1	Sialoadhesin, CD169	Clearance of Sialylated pathogens	Upregulation in ceroid lipofuscinosis (CLN)	Yes. Knockout mouse model exist	[34,82,104]
Siglec-2	CD22, B-Lymphocyte Cell Adhesion Molecule (BL-CAM)	Regulation of B cell response upon inflammation	Microglial phagocytosis in aging brains	Yes. Knockout mouse model exist.	[35,68,105,106]
Siglec-3	CD33, gp67, p67	Inhibition of pathogenesis and clearance of amyloid plaques	Implicated in LOAD (Late Onset of Alzheimer’s Disease)	Yes. Knockout mouse model exist but mouse and human CD33 are very different (explained in the text)	[14,27,90,94]
Siglec-4	Myelin associated glycoprotein (MAG)	Inhibition of neurite outgrowth and stimulation of axon growth cone collapse	Multiple sclerosis and benign monoclonal gammopathies	Yes. Knockout mouse model exist	[98,107,108,109]
Siglec-11	NA	Reduction of inflammation, Impairment of phagocytosis and decrease in microglial neurotoxicity	Autism, Schizophrenia and bipolar disorder (indirect evidence)	Yes. Humanized knock in mouse model exist.	[4,78,79,101,102,110]
Siglec-E	NA	Neuroprotection, Inhibition of phagocytosis of neural debris and regulation of reactive oxygen species (ROS) production	NA	Yes. Knock out mouse model and knowk in E16 mouse model exist.	[22,23,53,60]
Siglec-F	NA	Protection of neuronal integrity	Neurodegenerative diseases	Yes. Knockout mouse model exist	[63,111,112]
Siglec-H	NA	Enhancement of phagocytosis	Amyotrophic lateral sclerosis and brain tumor	Yes. Knockout mouse model exist.	[28,81,113]

## References

[1] Freeze H.H., Eklund E.A., Ng B.G., Patterson M.C. (2015). Neurological Aspects of Human Glycosylation Disorders. Annu. Rev. Neurosci..

[2] Scott H., Panin V.M., Yu R.K., Schengrund C.-L. (2009). N-Glycosylation in Regulation of the Nervous System. Glycobiology of the Nervous System.

[3] Varki A., Schnaar R.L., Schauer R., Varki A., Cummings R.D., Esko J.D. (2017). Sialic Acids and Other Nonulosonic Acids. Essentials of Glycobiology.

[4] Schnaar R.L., Gerardy-Schahn R., Hildebrandt H. (2014). Sialic Acids in the Brain: Gangliosides and Polysialic Acid in Nervous System Development, Stability, Disease, and Regeneration. Physiol. Rev..

[5] Pillai S., Netravali I.A., Cariappa A., Mattoo H. (2012). Siglecs and immune regulation. Annu. Rev. Immunol..

[6] Stamenkovic I., Seed B. (1990). The B-cell antigen CD22 mediates monocyte and erythrocyte adhesion. Nature.

[7] Stamenkovic I., Sgroi D., Aruffo A., Sy M.S., Anderson T. (1991). The B lymphocyte adhesion molecule CD22 interacts with leukocyte common antigen CD45RO on T cells and α2-6 sialyltransferase, CD75, on B cells. Cell.

[8] Crocker P., Kelm S., Dubois C., Martin B., McWilliam A., Shotton D., Paulson J., Gordon S. (1991). Purification and properties of sialoadhesin, a sialic acid-binding receptor of murine tissue macrophages. EMBO J..

[9] Crocker P., Mucklow S., Bouckson V., McWilliam A., Willis A., Gordon S., Milon G., Kelm S., Bradfield P. (1994). Sialoadhesin, a macrophage sialic acid binding receptor for haemopoietic cells with 17 immunoglobulin-like domains. EMBO J..

[10] Kelm S., Pelz A., Schauer R., Filbin M.T., Tang S., De Bellard M.-E., Schnaar R.L., Mahoney J.A., Hartnell A., Bradfield P. (1994). Sialoadhesin, myelin-associated glycoprotein and CD22 define a new family of sialic acid-dependent adhesion molecules of the immunoglobulin superfamily. Curr. Boil..

[11] Crocker P.R., Clark E.A., Filbin M., Gordon S., Jones Y., Kehrl J.H., Kelm H., Douarin N.L., Powell L., Roder J. (2012). Siglecs: A family of sialic-acid binding lectins. Glycobiology.

[12] Varki A., Angata T. (2006). Siglecs-The major subfamily of I-type lectins. Glycobiology.

[13] Crocker P.R., Varki A. (2001). Siglecs, sialic acids and innate immunity. Trends Immunol..

[14] Der Linden E.C.M.B.-V., Angata T., Reynolds S.A., Powell L.D., Hedrick S.M., Varki A. (2003). CD33/Siglec-3 Binding Specificity, Expression Pattern, and Consequences of Gene Deletion in Mice. Mol. Cell. Boil..

[15] Paulson J.C., Macauley M.S., Kawasaki N. (2012). Siglecs as sensors of self in innate and adaptive immune responses. Ann. New York Acad. Sci..

[16] Angata T., Hayakawa T., Yamanaka M., Varki A., Nakamura M. (2006). Discovery of Siglec-14, a novel sialic acid receptor undergoing concerted evolution with Siglec-5 in primates. FASEB J..

[17] Cao H., Lakner U., De Bono B., Traherne J.A., Trowsdale J., Barrow A.D. (2008). SIGLEC16encodes a DAP12-associated receptor expressed in macrophages that evolved from its inhibitory counterpartSIGLEC11and has functional and non-functional alleles in humans. Eur. J. Immunol..

[18] Kameda Y., Takahata M., Komatsu M., Mikuni S., Hatakeyama S., Shimizu T., Angata T., Kinjo M., Minami A., Iwasaki N. (2013). Siglec-15 Regulates Osteoclast Differentiation by Modulating RANKL-Induced Phosphatidylinositol 3-Kinase/Akt and Erk Pathways in Association With Signaling Adaptor DAP12. J. Bone Miner. Res..

[19] Takamiya R., Ohtsubo K., Takamatsu S., Taniguchi N., Angata T. (2013). The interaction between Siglec-15 and tumor-associated sialyl-Tn antigen enhances TGF-β secretion from monocytes/macrophages through the DAP12-Syk pathway. Glycobiology.

[20] Ishida-Kitagawa N., Tanaka K., Bao X., Kimura T., Miura T., Kitaoka Y., Hayashi K., Sato M., Maruoka M., Ogawa T. (2012). Siglec-15 Protein Regulates Formation of Functional Osteoclasts in Concert with DNAX-activating Protein of 12 kDa (DAP12). J. Boil. Chem..

[21] Ali S.R., Fong J.J., Carlin A.F., Busch T.D., Linden R., Angata T., Areschoug T., Parast M., Varki N., Murray J. (2014). Siglec-5 and Siglec-14 are polymorphic paired receptors that modulate neutrophil and amnion signaling responses to group B Streptococcus. J. Exp. Med..

[22] Schwarz F., Landig C.S., Siddiqui S., Secundino I., Olson J., Varki N., Nizet V., Varki A. (2017). Paired Siglec receptors generate opposite inflammatory responses to a human-specific pathogen. EMBO J..

[23] Stanczak M.A., Siddiqui S.S., Trefny M.P., Thommen D.S., Boligan K.F., Von Gunten S., Tzankov A., Tietze L., Lardinois D., Heinzelmann-Schwarz V. (2018). Self-associated molecular patterns mediate cancer immune evasion by engaging Siglecs on T cells. J. Clin. Investig..

[24] Brinkman-Van Der linden E.C.M., Hurtado-Ziola N., Hayakawa T., Wiggleton L., Benirschke K., Varki A., Varki N. (2007). Human-specific expression of Siglec-6 in the placenta. Glycobiology.

[25] Mitra N., Banda K., Altheide T.K., Schaffer L., Johnson-Pais T.L., Beuten J., Leach R.J., Angata T., Varki N., Varki A. (2011). SIGLEC12, a Human-specific Segregating (Pseudo)gene, Encodes a Signaling Molecule Expressed in Prostate Carcinomas. J. Boil. Chem..

[26] Dharmadhikari G., Stolz K., Hauke M., Morgan N.G., Varki A., De Koning E., Kelm S., Maedler K. (2017). Siglec-7 restores β-cell function and survival and reduces inflammation in pancreatic islets from patients with diabetes. Sci. Rep..

[27] Estus S., Shaw B.C., Devanney N., Katsumata Y., Press E.E., Fardo D.W. (2019). Evaluation of CD33 as a genetic risk factor for Alzheimer’s disease. Acta Neuropathol..

[28] Siew J.J., Chern Y. (2018). Microglial Lectins in Health and Neurological Diseases. Front. Mol. Neurosci..

[29] Linnartz-Gerlach B., Mathews M., Neumann H. (2014). Sensing the neuronal glycocalyx by glial sialic acid binding immunoglobulin-like lectins. Neuroscience.

[30] Linnartz-Gerlach B., Kopatz J., Neumann H. (2014). Siglec functions of microglia. Glycobiology.

[31] Landig C.S., Hazel A., Kellman B.P., Fong J.J., Schwarz F., Agarwal S., Varki N., Massari P., Lewis N.E., Ram S. (2019). Evolution of the exclusively human pathogen Neisseria gonorrhoeae: Human-specific engagement of immunoregulatory Siglecs. Evol. Appl..

[32] Tecle E., Reynoso H.S., Wang R., Gagneux P. (2019). The female reproductive tract contains multiple innate sialic acid-binding immunoglobulin-like lectins (Siglecs) that facilitate sperm survival. J. Boil. Chem..

[33] Hartnell A., Steel J., Turley H., Jones M., Jackson D.G., Crocker P.R. (2001). Characterization of human sialoadhesin, a sialic acid binding receptor expressed by resident and inflammatory macrophage populations. Blood.

[34] Crocker P.R., Hartnell A., Munday J., Nath D. (1997). The potential role of sialoadhesin as a macrophage recognition molecule in health and disease. Glycoconj. J..

[35] Jellusova J., Nitschke L. (2012). Regulation of B Cell Functions by the Sialic Acid-Binding Receptors Siglec-G and CD22. Front. Immunol..

[36] Andrews R.G., Torok-Storb B., Bernstein I.D. (1983). Myeloid-associated differentiation antigens on stem cells and their progeny identified by monoclonal antibodies. Blood.

[37] Freeman S.D., Kelm S., Barber E.K., Crocker P.R. (1995). Characterization of CD33 as a new member of the sialoadhesin family of cellular interaction molecules. Blood.

[38] Martínez-Esparza M., Miguel R.C.-S., Hernández-Caselles T., Pérez-Oliva A.B., Vicente-Fernández J.J., García-Peñarrubia P. (2011). Epitope mapping, expression and post-translational modifications of two isoforms of CD33 (CD33M and CD33m) on lymphoid and myeloid human cells. Glycobiol..

[39] Hernández-Caselles T., Martínez-Esparza M., Pérez-Oliva A.B., Quintanilla-Cecconi A.M., García-Alonso A., Alvarez-López D.M.R., García-Peñarrubia P. (2006). A study of CD33 (SIGLEC-3) antigen expression and function on activated human T and NK cells: Two isoforms of CD33 are generated by alternative splicing. J. Leukoc. Boil..

[40] Quarles R.H. (2002). Myelin sheaths: Glycoproteins involved in their formation, maintenance and degeneration. Cell. Mol. Life Sci..

[41] Quarles R.H., Everly J.L., Brady R.O. (1973). Evidence for the close association of a glycoprotein with myelin in rat brain. J. Neurochem..

[42] Arquint M., Roder J., Chia L.S., Down J., Wilkinson D., Bayley H., Braun P., Dunn R. (1987). Molecular cloning and primary structure of myelin-associated glycoprotein. Proc. Natl. Acad. Sci. USA.

[43] Sutcliffe J., Milner R.J., Shinnick T.M., Bloom F.E. (1983). Identifying the protein products of brain-specific genes with antibodies to chemically synthesized peptides. Cell.

[44] Vinson M., Strijbos P.J.L.M., Rowles A., Facci L., Moore S.E., Simmons D.L., Walsh F.S. (2001). Myelin-associated glycoprotein interacts with ganglioside GT1b. A mechanism for neurite outgrowth inhibition. J. Boil. Chem..

[45] Collins B.E., Yang L.J.-S., Mukhopadhyay G., Filbin M.T., Kiso M., Hasegawa A., Schnaar R.L. (1997). Sialic Acid Specificity of Myelin-associated Glycoprotein Binding. J. Boil. Chem..

[46] Trapp B.D. (1990). Myelin-Associated Glycoprotein Location and Potential Functions. Ann. N. Y. Acad. Sci..

[47] Angata T., Kerr S.C., Greaves D.R., Varki N.M., Crocker P.R., Varki A. (2002). Cloning and characterization of human Siglec-11: A recently evolved signaling molecule that can interact with SHP-1 and SHP-2 and is expressed by tissue macrophages, including brain microglia. J. Biol. Chem..

[48] Wang X., Mitra N., Cruz P., Deng L., Varki N., Angata T., Green E.D., Mullikin J., Hayakawa T., Varki A. (2012). Evolution of Siglec-11 and Siglec-16 Genes in Hominins. Mol. Boil. Evol..

[49] Hayakawa T., Angata T., Lewis A.L., Mikkelsen T.S., Varki N.M., Varki A. (2005). Evolution: A human-specific gene in microglia. Science.

[50] YU Z., MAOUI M., WU L., BANVILLE D., SHEN S.-H. (2015). mSiglec-E, a novel mouse CD33-related siglec (sialic acid-binding immunoglobulin-like lectin) that recruits Src homology 2 (SH2)-domain-containing protein tyrosine phosphatases SHP-1 and SHP-2. Biochem. J..

[51] Siddiqui S., Schwarz F., Springer S., Khedri Z., Yu H., Deng L., Verhagen A., Naito-Matsui Y., Jiang W., Kim D. (2017). Studies on the detection, expression, glycosylation, dimerization, and ligand binding properties of mouse Siglec-E. J. Biol. Chem..

[52] Zhang J.Q., Biedermann B., Nitschke L., Crocker P.R. (2004). The murine inhibitory receptor mSiglec-E is expressed broadly on cells of the innate immune system whereas mSiglec-F is restricted to eosinophils. Eur. J. Immunol..

[53] Uchiyama S., Sun J., Fukahori K., Ando N., Wu M., Schwarz F., Siddiqui S.S., Varki A., Marth J.D., Nizet V. (2019). Dual actions of group B Streptococcus capsular sialic acid provide resistance to platelet-mediated antimicrobial killing. Proc. Natl. Acad. Sci. USA.

[54] Spence S., Greene M.K., Fay F., Hams E., Saunders S.P., Hamid U., Fitzgerald M., Beck J., Bains B.K., Smyth P. (2015). Targeting Siglecs with a sialic acid-decorated nanoparticle abrogates inflammation. Sci. Transl. Med..

[55] Läubli H., Pearce O.M.T., Schwarz F., Siddiqui S.S., Deng L., Stanczak M.A., Deng L., Verhagen A., Secrest P., Lusk C. (2014). Engagement of myelomonocytic Siglecs by tumor-associated ligands modulates the innate immune response to cancer. Proc. Natl. Acad. Sci. USA.

[56] Boyd C.R., Orr S.J., Spence S., Burrows J.F., Elliott J., Carroll H.P., Brennan K., Ni Gabhann J., Coulter W.A., Johnston J.A. (2010). Siglec-E is up-regulated and phosphorylated following lipopolysaccharide stimulation in order to limit TLR-driven cytokine production. J. Immunol..

[57] Chen G.-Y., Brown N.K., Wu W., Khedri Z., Yu H., Chen X., van de Vlekkert D., D’Azzo A., Zheng P., Liu Y. (2014). Broad and direct interaction between TLR and Siglec families of pattern recognition receptors and its regulation by Neu1. Elife.

[58] McMillan S.J., Sharma R.S., McKenzie E.J., Richards H.E., Zhang J., Prescott A., Crocker P.R. (2013). Siglec-E is a negative regulator of acute pulmonary neutrophil inflammation and suppresses CD11b b2-integrin-dependent signaling. Blood.

[59] Schwarz F., Pearce O.M., Wang X., Samraj A.N., Läubli H., Garcia J.O., Lin H., Fu X., Garcia-Bingman A., Secrest P. (2015). Siglec receptors impact mammalian lifespan by modulating oxidative stress. Elife.

[60] Claude J., Linnartz-Gerlach B., Kudin A.P., Kunz W.S., Neumann H. (2013). Microglial CD33-Related Siglec-E Inhibits Neurotoxicity by Preventing the Phagocytosis-Associated Oxidative Burst. J. Neurosci..

[61] Angata T. (2001). Cloning and Characterization of a Novel Mouse Siglec, mSiglec-F. DIFFERENTIAL EVOLUTION OF THE MOUSE AND HUMAN (CD33) Siglec-3-RELATED GENE CLUSTERS. J. Boil. Chem..

[62] Tateno H., Crocker P.R., Paulson J.C. (2005). Mouse Siglec-F and human Siglec-8 are functionally convergent paralogs that are selectively expressed on eosinophils and recognize 6′-sulfo-sialyl Lewis X as a preferred glycan ligand. Glycobiology.

[63] Wielgat P., Braszko J.J. (2012). The participation of sialic acids in microglia–neuron interactions. Cell. Immunol..

[64] Blasius A.L., Colonna M. (2006). Sampling and signaling in plasmacytoid dendritic cells: The potential roles of Siglec-H. Trends Immunol..

[65] Konishi H., Kobayashi M., Kunisawa T., Imai K., Sayo A., Malissen B., Crocker P.R., Sato K., Kiyama H. (2017). Siglec-H is a microglia-specific marker that discriminates microglia from CNS-associated macrophages and CNS-infiltrating monocytes. Glia.

[66] Perry V.H., Crocker P.R., Gordon S. (1992). The blood–brain barrier regulates the expression of a macrophage sialic acid-binding receptor on microglia. J. Cell Sci..

[67] Mott R.T., Ait-Ghezala G., Town T., Mori T., Vendrame M., Zeng J., Ehrhart J., Mullan M., Tan J., Mullan M. (2004). Neuronal expression of CD22: Novel mechanism for inhibiting microglial proinflammatory cytokine production. Glia.

[68] Pluvinage J.V., Haney M.S., Smith B.A.H., Sun J., Iram T., Bonanno L., Li L., Lee D.P., Morgens D.W., Yang A.C. (2019). CD22 blockade restores homeostatic microglial phagocytosis in ageing brains. Nature.

[69] Johnson P.W., Abramow-Newerly W., Seilheimer B., Sadoul R., Tropak M.B., Arquint M., Dunn R.J., Schachner M., Roder J.C. (1989). Recombinant myelin-associated glycoprotein confers neural adhesion and neurite outgrowth function. Neuron.

[70] Turnley A.M., Bartlett P.F. (1998). MAG and MOG enhance neurite outgrowth of embryonic mouse spinal cord neurons. NeuroReport.

[71] Mukhopadhyay G., Doherty P., Walsh F.S., Crocker P.R., Filbin M.T. (1994). A novel role for myelin-associated glycoprotein as an inhibitor of axonal regeneration. Neuron.

[72] Quarles R.H. (2007). Myelin-associated glycoprotein (MAG): Past, present and beyond. J. Neurochem..

[73] Cai D., Qiu J., Cao Z., McAtee M., Bregman B.S., Filbin M.T. (2001). Neuronal Cyclic AMP Controls the Developmental Loss in Ability of Axons to Regenerate. J. Neurosci..

[74] McKerracher L., Rosen K.M. (2015). MAG, myelin and overcoming growth inhibition in the CNS. Front. Mol. Neurosci..

[75] Bartsch U., Bandtlow C.E., Schnell L., Bartsch S., Spillmann A.A., Rubin B.P., Hillenbrand R., Schwab D.M.E., Schachner M. (1995). Lack of evidence that myelin-associated glycoprotein is a major inhibitor of axonal regeneration in the CNS. Neuron.

[76] Tang S., Woodhall R.W., Shen Y.J., Debellard M.E., Saffell J.L., Doherty P., Walsh F.S., Filbin M.T. (1997). Soluble myelin-associated glycoprotein (MAG) found in vivo inhibits axonal regeneration. Mol. Cell. Neurosci..

[77] Tang S., Qiu J., Nikulina E., Filbin M.T. (2001). Soluble Myelin-Associated Glycoprotein Released from Damaged White Matter Inhibits Axonal Regeneration. Mol. Cell. Neurosci..

[78] Linnartz B., Wang Y., Neumann H. (2010). Microglial Immunoreceptor Tyrosine-Based Activation and Inhibition Motif Signaling in Neuroinflammation. Int. J. Alzheimer’s Dis..

[79] Wang Y., Neumann H. (2010). Alleviation of Neurotoxicity by Microglial Human Siglec-11. J. Neurosci..

[80] Pshezhetsky A.V., Ashmarina M. (2018). Keeping it trim: Roles of neuraminidases in CNS function. Glycoconj. J..

[81] Kopatz J., Beutner C., Welle K., Bodea L.-G., Reinhardt J., Claude J., Linnartz-Gerlach B., Neumann H., Linnartz-Gerlach B. (2013). Siglec-h on activated microglia for recognition and engulfment of glioma cells. Glia.

[82] Groh J., Ribechini E., Stadler D., Schilling T., Lutz M.B., Martini R. (2016). Sialoadhesin promotes neuroinflammation-related disease progression in two mouse models of CLN disease. Glia.

[83] Mabbott N. (2017). How do PrPSc Prions Spread between Host Species, and within Hosts?. Pathogens.

[84] Bradford B.M., Crocker P.R., Mabbott N.A. (2014). Peripheral prion disease pathogenesis is unaltered in the absence of sialoadhesin (Siglec-1/CD169). Immunology.

[85] Ip C.W., Kroner A., Crocker P.R., Nave K.-A., Martini R. (2007). Sialoadhesin deficiency ameliorates myelin degeneration and axonopathic changes in the CNS of PLP overexpressing mice. Neurobiol. Dis..

[86] Bertram L., Lange C., Mullin K., Parkinson M., Hsiao M., Hogan M.F., Schjeide B.M.M., Hooli B., DiVito J., Ionita I. (2008). Genome-wide Association Analysis Reveals Putative Alzheimer’s Disease Susceptibility Loci in Addition to APOE. Am. J. Hum. Genet..

[87] Hollingworth P., Harold D., Sims R., Gerrish A., Lambert J.C., Carrasquillo M.M., Abraham R., Hamshere M.L., Pahwa J.S., Moskvina V. (2011). Common variants at ABCA7, MS4A6A/MS4A4E, EPHA1, CD33 and CD2AP are associated with Alzheimer’s disease. Nat. Genet..

[88] Walker D.G., Whetzel A.M., Serrano G., Sue L.I., Beach T.G., Lue L.F. (2015). Association of CD33 polymorphism rs3865444 with Alzheimer’s disease pathology and CD33 expression in human cerebral cortex. Neurobiol. Aging.

[89] Naj A.C., Jun G., Beecham G.W., Wang L.S., Vardarajan B.N., Buros J., Gallins P.J., Buxbaum J.D., Jarvik G.P., Crane P.K. (2011). Common variants at MS4A4/MS4A6E, CD2AP, CD33 and EPHA1 are associated with late-onset Alzheimer’s disease. Nat. Genet..

[90] Griciuc A., Serrano-Pozo A., Parrado A.R., Lesinski A.N., Asselin C.N., Mullin K., Hooli B., Choi S.H., Hyman B.T., Tanzi R.E. (2013). Alzheimer’s Disease Risk Gene CD33 Inhibits Microglial Uptake of Amyloid Beta. Neuron.

[91] Malik M., Simpson J.F., Parikh I., Wilfred B.R., Fardo D.W., Nelson P.T., Estus S. (2013). CD33 Alzheimer’s Risk-Altering Polymorphism, CD33 Expression, and Exon 2 Splicing. J. Neurosci..

[92] Raj T., Ryan K.J., Replogle J.M., Chibnik L.B., Rosenkrantz L., Tang A., Rothamel K., Stranger B.E., Bennett D.A., Evans D.A. (2014). CD33: Increased inclusion of exon 2 implicates the Ig V-set domain in Alzheimer’s disease susceptibility. Hum. Mol. Genet..

[93] Bradshaw E.M., Chibnik L.B., Keenan B.T., Ottoboni L., Raj T., Tang A., Rosenkrantz L.L., Imboywa S., Lee M., Von Korff A. (2013). CD33 Alzheimer’s disease locus: Altered monocyte function and amyloid biology. Nat. Neurosci..

[94] Siddiqui S.S., Springer S.A., Verhagen A., Sundaramurthy V., Alisson-Silva F., Jiang W., Ghosh P., Varki A. (2017). The Alzheimer’s Disease–protective CD33 splice variant mediates adaptive loss of function via diversion to an intracellular pool. J. Biol. Chem..

[95] Fruttiger M., Montag D., Schachner M., Martini R. (1995). Crucial Role for the Myelin-associated Glycoprotein in the Maintenance of Axon-Myelin Integrity. Eur. J. Neurosci..

[96] Bjartmar C., Trapp B.D. (2001). Axonal and neuronal degeneration in multiple sclerosis: Mechanisms and functional consequences. Curr. Opin. Neurol..

[97] Barbay S., Plautz E.J., Zoubina E., Frost S.B., Cramer S.C., Nudo R.J. (2015). Effects of Postinfarct Myelin-Associated Glycoprotein Antibody Treatment on Motor Recovery and Motor Map Plasticity in Squirrel Monkeys. Stroke.

[98] Dalakas M.C. (2018). Advances in the diagnosis, immunopathogenesis and therapies of IgM-anti-MAG antibody-mediated neuropathies. Ther. Adv. Neurol. Disord..

[99] Ilyas A.A., Quarles R.H., MacIntosh T.D., Dobersen M.J., Trapp B.D., Dalakas M.C., Brady R.O. (1984). IgM in a human neuropathy related to paraproteinemia binds to a carbohydrate determinant in the myelin-associated glycoprotein and to a ganglioside. Proc. Natl. Acad. Sci. USA.

[100] Dalakas M.C. (2010). Pathogenesis and Treatment of Anti-MAG Neuropathy. Curr. Treat. Options Neurol..

[101] Brennaman L.H., Maness P.F., Berezin V. (2009). NCAM in Neuropsychiatric and Neurodegenerative Disorders. Structure and Function of the Neural Cell Adhesion Molecule NCAM.

[102] Vawter M.P. (2000). Dysregulation of the neural cell adhesion molecule and neuropsychiatric disorders. Eur. J. Pharmacol..

[103] Pham M.T., Pollock K.M., Rose M.D., Cary W.A., Stewart H.R., Zhou P., Nolta J.A., Waldau B. (2018). Generation of human vascularized brain organoids. NeuroReport.

[104] Oetke C., Vinson M.C., Jones C., Crocker P.R. (2006). Sialoadhesin-Deficient Mice Exhibit Subtle Changes in B- and T-Cell Populations and Reduced Immunoglobulin M Levels. Mol. Cell. Boil..

[105] Nitschke L., Carsetti R., Ocker B., Köhler G., Lamers M.C. (1997). CD22 is a negative regulator of B-cell receptor signalling. Curr. Boil..

[106] Samardzic T., Marinkovic D., Danzer C.P., Gerlach J., Nitschke L., Wirth T. (2002). Reduction of marginal zone B cells in CD22-deficient mice. Eur. J. Immunol..

[107] Li M., Shibata A., Li C., Braun P.E., McKerracher L., Roder J., Kater S.B., David S. (1996). Myelin-associated glycoprotein inhibits neurite/axon growth and causes growth cone collapse. J. Neurosci. Res..

[108] DeBellard M.-E., Tang S., Mukhopadhyay G., Shen Y.-J., Filbin M.T. (1996). Myelin-Associated Glycoprotein Inhibits Axonal Regeneration from a Variety of Neurons via Interaction with a Sialoglycoprotein. Mol. Cell. Neurosci..

[109] Montag D., Giese K.P., Bartsch U., Martini R., Lang Y., Blüthmann H., Karthigasan J., Kirschner D.A., Wintergerst E.S., Nave K.-A. (1994). Mice deficient for the glycoprotein show subtle abnormalities in myelin. Neuron.

[110] Karlstetter M., Kopatz J., Aslanidis A., Shahraz A., Caramoy A., Linnartz-Gerlach B., Lin Y., Lückoff A., Fauser S., Düker K. (2017). Polysialic acid blocks mononuclear phagocyte reactivity, inhibits complement activation, and protects from vascular damage in the retina. EMBO Mol. Med..

[111] Zhang M., Angata T., Cho J.Y., Miller M., Broide D.H., Varki A. (2007). Defining the in vivo function of Siglec-F, a CD33-related Siglec expressed on mouse eosinophils. Blood.

[112] McMillan S.J., Richards H.E., Crocker P.R. (2014). Siglec-F-dependent negative regulation of allergen-induced eosinophilia depends critically on the experimental model. Immunol. Lett..

[113] Schmitt H., Sell S., Koch J., Seefried M., Sonnewald S., Daniel C., Winkler T.H., Nitschke L. (2016). Siglec-H protects from virus-triggered severe systemic autoimmunity. J. Exp. Med..

